# Problems with the upper limb in arthrogryposis

**DOI:** 10.1007/s11832-015-0694-4

**Published:** 2015-10-19

**Authors:** Ruth Lester

**Affiliations:** Childrens Hand and Upper Limb Service, Birmingham Childrens Hospital, Steelhouse Lane, Birmngham, B4 6NH UK; 18 Church Road, Edgbaston, Birmingham, B15 3TA UK

**Keywords:** Upper limb, Arthrogryposis, Soft tissue release, Thumb web

## Abstract

**Introduction:**

This short paper presents a personal view of the thinking processes around the assessment and development of individualised management plans for dealing with the problems of the upper limb in Arthrogryposis in children.

**Background:**

The paper will offer a definition of arthrogryposis, its incidence and the range of anomalies. The problems associated with joint contractures are defined. The goals of treatment include improvement in function as well as cosmesis.

**Priorities and goals of treatment:**

Managing the mobility of the whole child is very important in deciding what should be offered surgically in the upper limb. Early manipulation and stretching as well as the pros and cons of splints are discussed.

**Principles of surgical procedures:**

The principles of surgery including soft tissue release, changing the position of the arc of movement of a joint, fixing stiff joints, tendon transfers and muscle transfers are presented. The value of thumb web release and the surgical procedures commonly used are described.

## Introduction

This paper presents a personal view of the thinking processes relating to the assessment and development of individualised management plans for dealing with the problems of the upper limb in children with arthrogryposis.

It is based on 20 years of personal experience of managing these children in a multidisciplinary environment through dedicated clinics at Birmingham Children’s Hospital NHS Foundation Trust which provides services for a population of 5.8 million in the West Midlands region and beyond.

## Definition

The word ‘arthrogryposis’ is a descriptive term meaning ‘curved’ (gryp) ‘joint’ (arthro).

A contracture in a specific joint implies limitation of movement of that joint.

Arthrogryposis can be defined as a congenital, nonprogressive limitation of movement of two or more joints in different areas of the body.

## Range of anomalies

Arthrogryposis is rare, occurring in 1 in 3,000 live births; however, at least one in every 200 infants is born with some type of congenital contracture or stiffness of a joint.

**Fig. 1:**
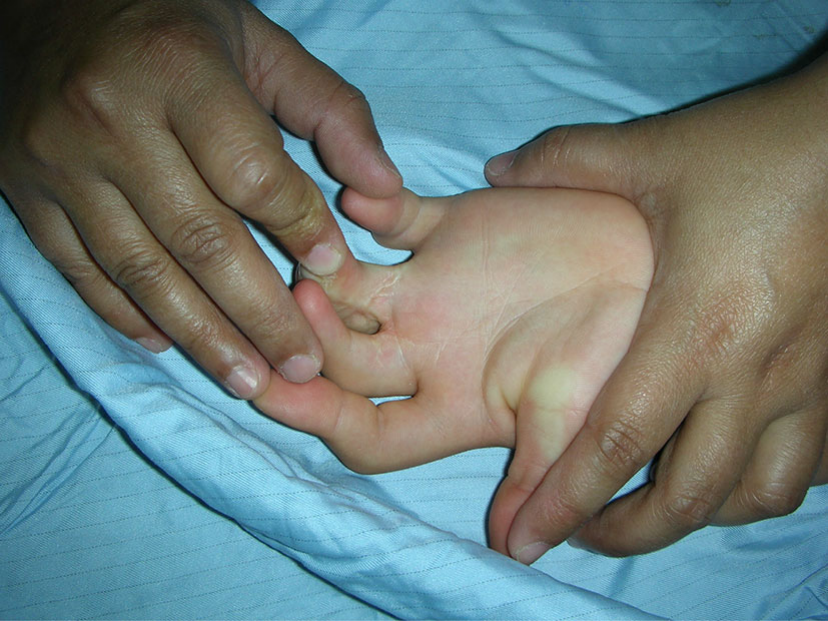
Camptodacyly of the fingers
- Thumb in palm isolated deformity or in association with distal arthrogryposis or arthrogryposis multiplex congenita (AMC) (see Fig. 2).

Clinical presentations to the upper limb surgeon are varied and can include:

- Camptodactyly of one or more fingers (see Fig. 1).

**Fig. 2:**
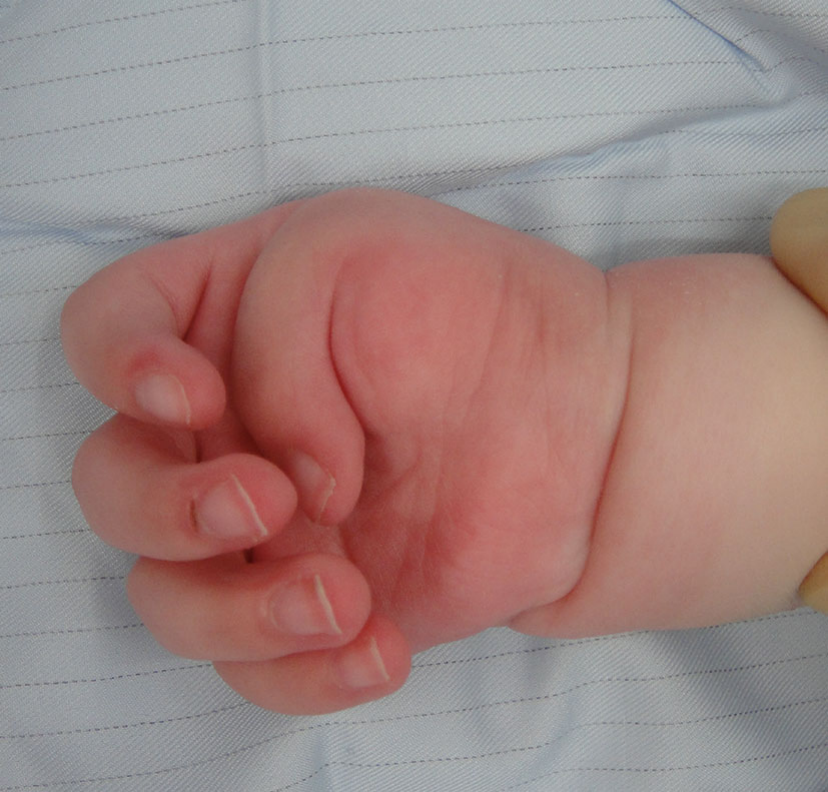
Thumb in palm deformity
- Symphalangism of one or more fingers (see Fig. 3).

**Fig. 3:**
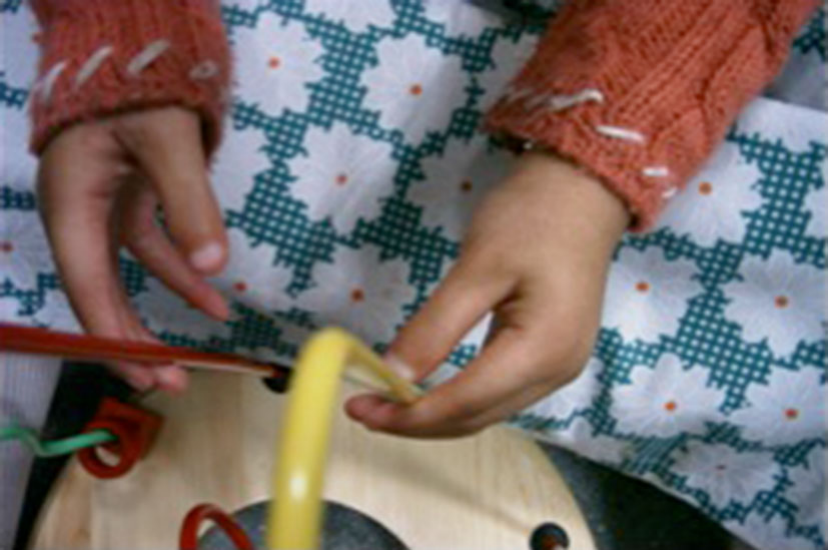
Symphalangism
- Distal arthrogryposis involving wrists, hands, ankles and feet (see Fig. 4).

**Fig. 4:**
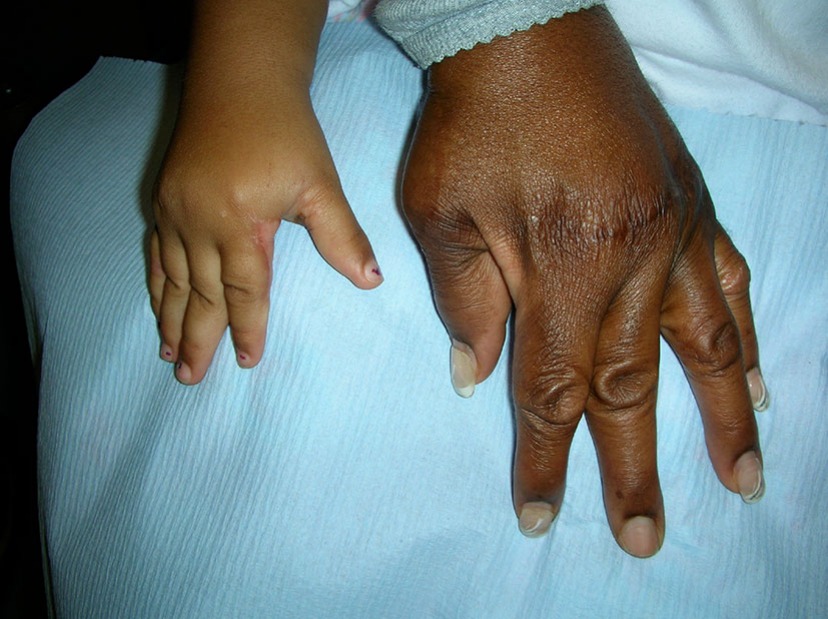
Distal arthrogryposis in a mother and daughter; the mother had MCP realignment and the child had thumb web release
- Single joint contractures (pterygium).
- Multiple joint contractures (AMC) (see Fig. 5).

**Fig. 5:**
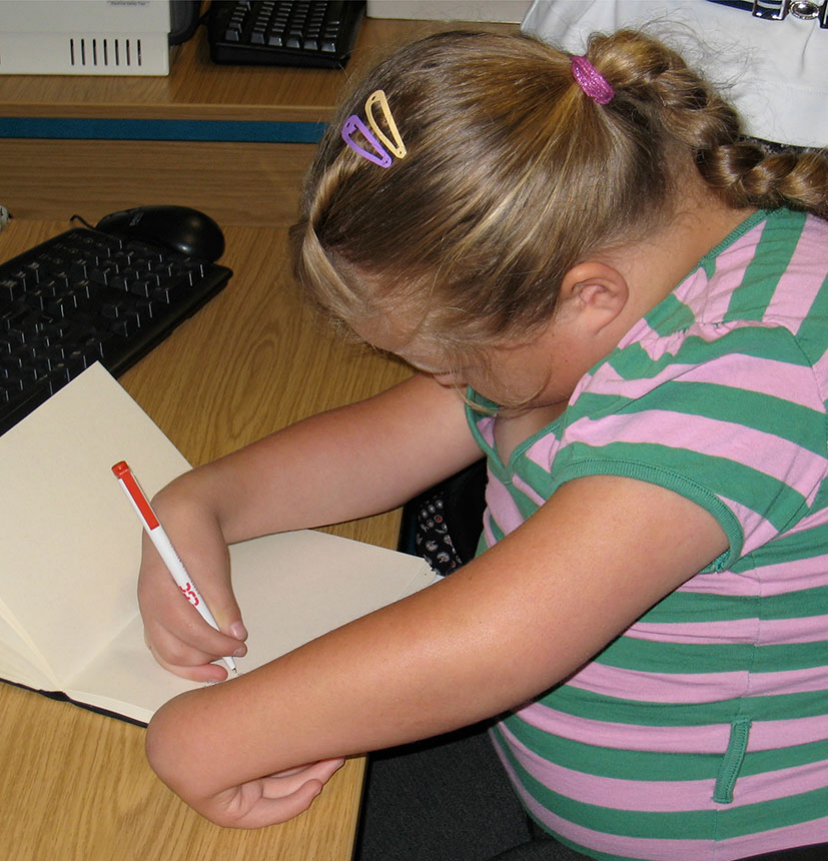
Arthrogryposis multiplex involving the upper limbs
- Multiple joint contractures with central nervous system involvement (usually lethal).
- Joint contractures associated with chromosomal disorders.

## The problems associated with joint contractures are

- Shortage of skin usually both longitudinally and horizontally.
- Short tendons.
- Joint stiffness, malposition and instability.
- Weak muscles (amyoplasia).

The priorities in assessing the upper limb in childrens with arthrogryposis include an examination of the shoulder, elbow and wrist as well as the thumb and fingers.

The goals of treatment include improvement in function as well as cosmesis.

The basic functions of the upper limb, which enable independent living, require the child to be able to get one hand to the mouth for feeding and one hand to reach the anus for toileting.

The ability to use a keyboard is a further vital skill for independent living.

In AMC the assessment of the whole child’s mobility issues becomes very important. The upper limbs will also be needed to manage walkers, crutches and wheelchairs in different ways. A significant percentage of these children are highly intelligent and very well motivated to achieve the ability to be independent and follow successful careers with minimal surgical intervention to the upper limbs.

## Principles of management

In the first 1–2 years of life there is likely to be a significant improvement in the range of movements of affected joints which can be further improved with manipulation and stretching. The parents need the support of trained physiotherapists to stretch and manipulate all affected joints on a regular basis several times a day, e.g., with each nappy change.

Splints are very difficult to fit in babies but may be helpful to continue the stretching during the night. Unrestricted free use of the upper limbs during the day is essential to allow the child to explore and find his/her own way of managing activities.

Occupational therapists can assist in providing orthoses or adaptive equipment to support some activities, e.g., specially shaped pens or cutlery as the child gets older.

Surgery to skin, soft tissue, joints, tendons and muscle has some value, but each patient’s surgical management plan needs to be individually and carefully thought through so that no existing or potential function is diminished. The timing of surgery is controversial and again any decisions need to be made after a thorough multidisiciplinary assessment of existing and potential functions. A stretching regime needs to be established before surgery is contemplated and then continued after surgery.

## Principles of surgical procedures

**Fig. 6:**
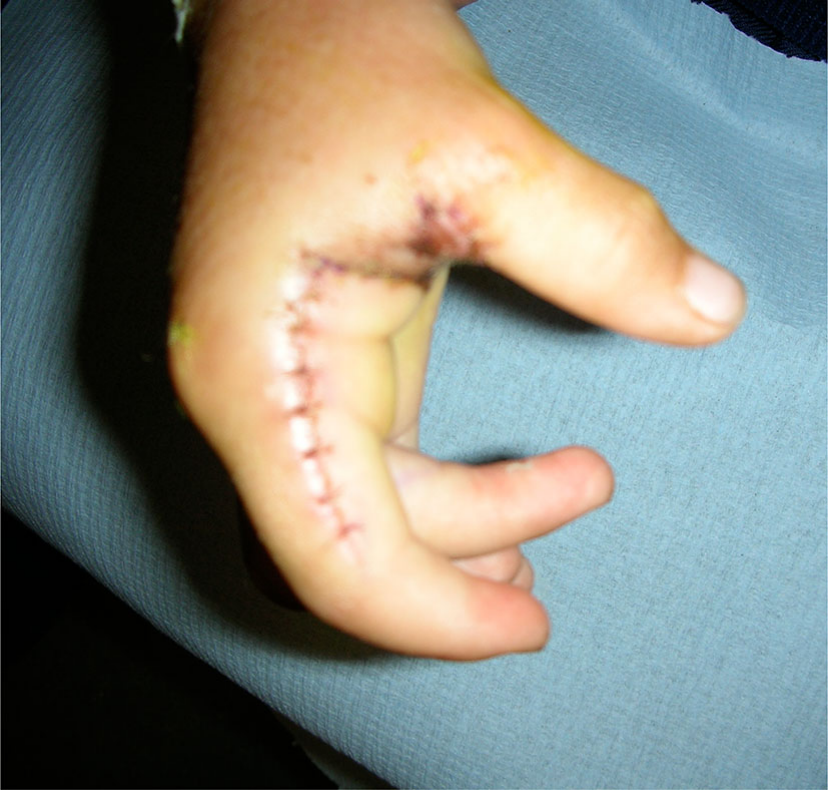
Flap from radial index used to deepen the thumb web

Soft tissue release of joint contractures associated with a programme of stretching and splintage can achieve a useful but modest increase in the range of movement of some joints such as the elbow or shoulder (Fig. 6).

In the proximal interphalangeal joints of the fingers, and metacarpophalangeal (MCP) joints of the thumb, further release of the volar plate can enhance the range of movement of these joints but only in a cooperative patient who will tolerate splints and exercises for a minimum of 6 months postoperatively. There is a high risk of development of a worse contracture if a postoperative splint and physiotherapy regime is not adhered to.

Possible soft tissue type release techniques include:

- Local flaps/*Z*-plasty flaps with or without additional full-thickness skin grafts.
- Tendon lengthening at the musculotendinous junction.

**Fig. 7:**
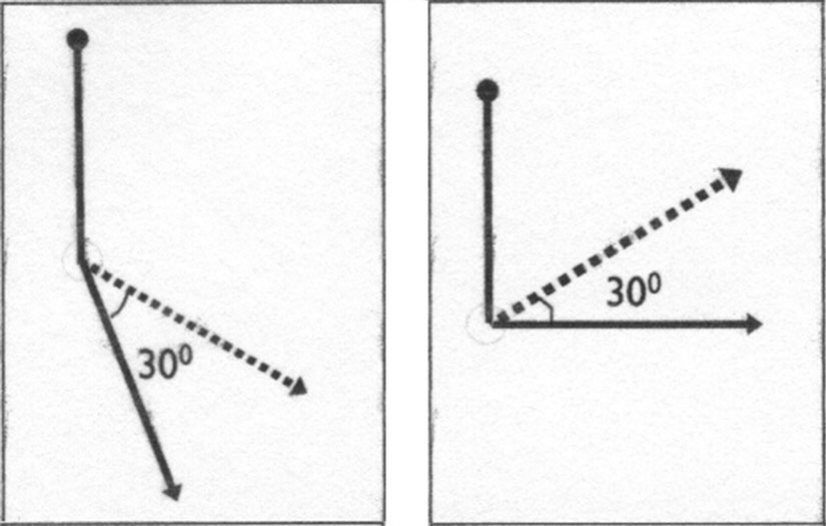
Changing the arc of movement position of a joint, e.g., elbow

Changing the position of the arc of movement of a joint may enable improved function, e.g., allowing movement of the elbow from 120 to 90º enables hand to mouth where as 180 to 150º does not (see Fig. 7). This can be achieved surgically by releasing joints as described above, or by carrying out wedge osteotomies near joints.

**Fig. 8:**
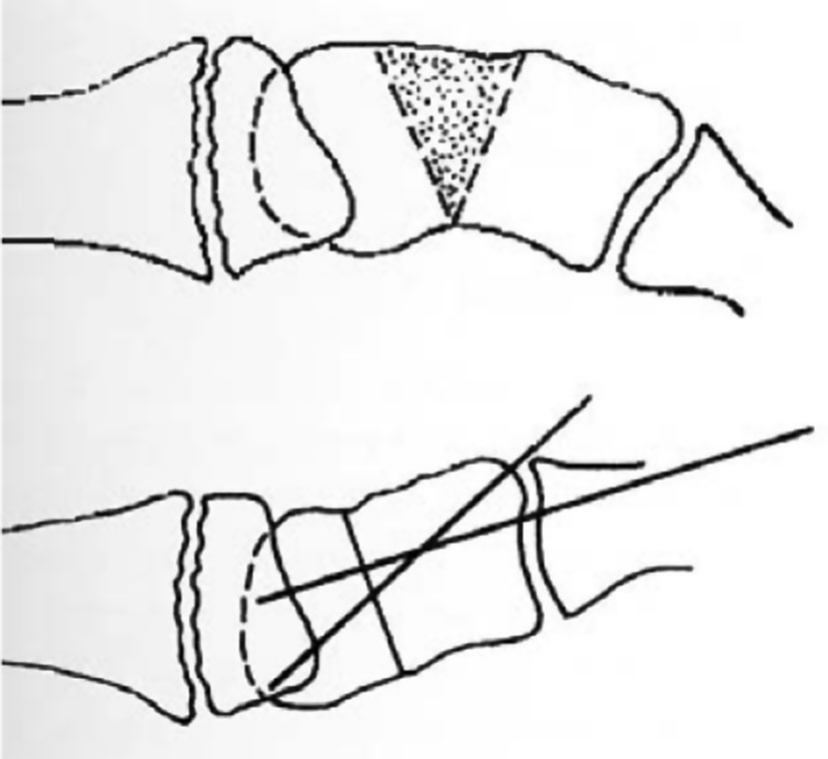
Schematic drawing of dorsal wedge osteotomy carpus

Fixing stiff joints into a position of function by arthrodesis may enable better function of the limb, e.g., bringing the wrist from a flexed position to neutral, may enable better finger function (see Fig. 8). The flexor tendons may function more efficiently if they are made proportionately slightly longer. Effective tendon lengthening can be achieved by bone shortening, which inevitably occurs during arthrodesis.

Tendon transfers may be of value in restoring a small range of active movement and improving the functional position of a joint if the passive range of joint movement is more than the active range.

This can be particularly useful in a younger child as a temporising procedure until skeletal maturity is reached and a definitive arthrodesis can be performed.

Transferring muscles for increasing active range of joint movement can be used only cautiously as further joint contractures frequently result.

Possible muscle transfers include:

- Triceps to biceps (long head of triceps wrapped round the ulna using a fascial graft to extend the tendon).
- Latissimus dorsi or pectoralis major to support elbow flexion (in these transfers care must be taken to ensure that further loss of function does not occur around the shoulder).
- Free muscle transfer, e.g., gracilis to restore elbow flexion (in this situation, a motor nerve has to be found for innervation of the transferred muscle).

### The stiff shoulder

We have very little experience in managing the internally rotated stiff shoulder but a humeral rotation osteotomy may help to reposition the arm in a better functional position.

### Thumb web release

This specific operation can be a valuable procedure where the ‘clasp thumb’ does not improve in the first year or so of life. The following surgical procedures are used as appropriate:

- Skin and soft tissue release within the thumb web by transferring a flap from the radial aspect of the index finger with or without additional full thickness skin grafts. This may involve detachment of the adductor pollicis distally as well as division of the fascia over the first dorsal interosseus down to the carpometacarpal joint (see Fig. 6).
- ‘*Z*’ lengthening of the flexor pollicis longus at the wrist at the musculotendinous junction.
- Volar plate release and/or arthrodesis or chondrodesis of the first MCP joint.

## Summary

The independence in managing activities of daily living in a patient with AMC is mainly dependant on the level of function achieved in the upper limbs.

Minimal functional requirements for independence include the ability to get hand to mouth and hand to bottom for toileting.

The mainstay of management for the upper limb has to be physiotherapy, occupational therapy and the use of orthoses.

The surgical approach to joint contractures of the upper limb needs to be cautious. A thorough multidisciplinary assessment of the patient has to be made preoperatively. Access to postoperative rehabilitation services is crucial to improving the outcomes after surgery.

A variety of surgical procedures using the above-outlined principles may be helpful but each case needs to be individually assessed.

The above opinions are personal but have been influenced by discussions with my colleagues from all over the world. Van Heest et al. [1] and Mennen et al. [2] are suggested as further reading.
